# Home and health among different sub-groups of the ageing population: a comparison of two cohorts living in ordinary housing in Sweden

**DOI:** 10.1186/s12877-016-0265-7

**Published:** 2016-04-26

**Authors:** Henrik Ekström, Steven M. Schmidt, Susanne Iwarsson

**Affiliations:** Department of Health Sciences, Lund University, P.O. Box 157, Lund, SE-221 00 Sweden

**Keywords:** Aspects of home, Health, Younger older, Very old

## Abstract

**Background:**

At present a majority of older people remain in their ordinary homes. Research has generated knowledge about home and health dynamics and increased the awareness of the complexity of housing as related to ageing. As this knowledge is based mainly on research on very old, single-living people in ordinary housing there is a need to study other sub-groups of the ageing population. Thus, the aim of the present descriptive study was to compare a younger old cohort with a very old cohort living in ordinary housing in Sweden in order to shed new light on home and health dynamics in different sub-groups of the ageing population.

**Methods:**

Cross-sectional study of two population-based cohorts: one aged 67–70 years (*n =* 371) and one aged 79–89 years (*n =* 397) drawn from existing Swedish databases. Structured interviews and observations were conducted to collect data about socio-demographics, aspects of home, and symptoms. Besides descriptive statistics we computed tests of differences using the Chi-squared test and Mann–Whitney U-test.

**Results:**

Accessibility was significantly lower in the very old cohort compared to the younger old cohort even though the former were objectively assessed to have fewer environmental barriers. Those in the very old cohort perceived aspects of their housing situation as worse and were more dependent on external influences managing their housing situation. Although a larger proportion of the very old cohort had more functional limitations 22% were independent in ADL. In the younger old cohort 17% were dependent in ADL.

**Conclusions:**

Keeping in mind that there were cohort differences beyond that of age, despite fewer environmental barriers in their dwellings the very old community-living cohort lived in housing with more accessibility problems compared to those of the younger old cohort, caused by their higher prevalence of functional limitations. Those in the very old cohort perceived themselves in a less favourable situation, but still as satisfied with housing as those in the younger old cohort. This kind of knowledge is indicative for prevention and intervention in health care and social services as well as for housing provision and societal planning. Further studies based on truly comparable cohorts are warranted.

## Background

Housing in old age is a burning issue throughout the world, and there is a great need for research on the home and health situation in different groups of the ageing population. Overall, as a result of policy changes in the Western world there has been a shift from institutional care to home health services, and the majority of people remains in their ordinary homes well into old age [1, 2]. In Sweden, the majority of older people live in the same types of ordinary housing as the younger population [2]. Also among very old people, only a small percentage resides in special housing. Against this background, the public debate raises concerns about how to supply the optimal type of housing to senior citizens with different functional statuses and different needs [3]. A first step would be to refine the discussion based on research comparing the home and health situations of different sub-groups of the ageing population rather than considering all older people as a homogeneous collective. Accordingly, there is a need for more nuanced and sub-group based housing policies and practices to meet the needs on an individual level, but the current scientific evidence is insufficient to come up with effective solutions regarding the home and health situation along the ageing process.

Most studies on ageing cover a multitude of information on the ageing person, but empirical ageing research with a balanced view on person and environment remains rare. Within the gerontology literature there is consensus that both personal and environmental resources contribute to healthy ageing. Nevertheless, the contribution of key elements of the immediate environment, such as the home, remains overlooked. As put forward by Wahl et. al., [4], there is a need for a deepened understanding of the interchange between people and their environments. During the last decade, our research has influenced the international knowledge frontier regarding home and health dynamics and increased the awareness of the complexity of housing related to ageing (see e.g., [5]). Recently, synthesizing 35 original articles from the ENABLE-AGE project we highlighted that in very old age, the interplay between aspects of perceived and objective housing and aspects of health could influence residential decision-making (), independence in daily activities and social participation [6]. As yet, this knowledge is based on longitudinal research on very old, single-living people in five European countries [7], and thus there is also a need to study other sub-groups in the ageing population.

Studies on home and health dynamics often relate to Lawton and Nahemow’s [8] ecological theory of ageing (ETA) and subsequent elaborations such as those presented by Scheidt and Norris-Baker [9]. According to the ETA, the interacting combination of an individual’s competence and the demands of the environment (person-environment fit; P-E-fit) are important for an individual’s adaptive behavior and level of functioning. Moreover, the docility hypothesis suggested that the lower an individual’s competence, the greater the impact of the environment on his or her ability to compensate for the negative consequences of environmental press. Most important, the environment should be understood as a dynamic and context-bound phenomenon which encompasses aspects of objective as well as perceived character [10].

It has long been argued that there is a need for a broader diversity of research regarding home environments including different sub-groups of the ageing population with different levels of competencies and life experiences [1]. In response to this quest, in a recent study on people aged 67–70 living in ordinary housing in south Sweden [10], we showed that the majority are in good health and have few functional limitations. Women have more functional limitations and report more symptoms than men. While environmental barriers do exist in all dwellings (in particular in kitchens and hygiene rooms), on an overall level they are more common in multi-family than in single-family dwellings. Based on analyses made for a recent Swedish governmental commission [3] we also know that the environmental barriers in multi-family dwellings are of a somewhat different character than those in single-family dwellings, and that there are significant differences between housing types depending on building period.

In order to expose contrasts and shed new light on home and health dynamics in different sub-groups of the ageing population, the aim of the present descriptive study was to compare a younger old cohort to a very old cohort living in ordinary housing in Sweden.

## Methods

### Participants and procedures

In the present cross-sectional comparison of two cohorts representing different sub-groups of the ageing population, participants were drawn from the ENABLE-AGE Survey Study (very old cohort) [11], and from the Home and Health in the Third Age Study (younger old cohort) [12]. Data from ENABLE-AGE (Swedish sub-sample) was from the baseline data collection completed in 2003. The younger old participants were a subsample of the Good Ageing in Skåne (GÅS) Project, which is part of the Swedish National Study on Ageing and Care (SNAC) [13, 14]. Data was collected during an eight-month period in 2010–2011.

The two groups are described in Table 1. The younger old and the very old age cohorts consisted of 371 and 397 participants, respectively. In the younger old cohort the median age was 68 (q1 = 67, q3 = 69) years with a majority of women (57.1%). In the very old cohort the median age was 84 years (q1 = 81, q3 = 87), and the proportion of women was 74.6%. Participants in the younger old cohort were drawn from a general population without any attention to the type of area where they lived, while the recruitment of the very old cohort was focused on single-living people in urban areas, which accounts for differences seen in some of the demographic variables. All participants were community-dwelling at the time of data collection.

**Table 1:** Description of the younger old and very old cohort

Characteristic	Younger old cohort *N =* 371	Very old cohort *N =* 397
Age md (q1-q3)	68 (67–69)	84 (81–87)
Sex		
Women % (*n*)	57.1 (212)	74.6 (296)
Men % (*n*)	42.9 (159)	25.4 (101)
Marital status		
Living with a partner % (*n*)	64.2 (238)	0 (0)
Single or relation to a partner	3.2 (12)	4.3 (17)
Living alone % (*n*)	32.3 (120)	95.7(380)
Education % (*n*)		
Unfinished elementary school % (*n*)	3.0 (11)	0.3 (1)
Elementary school % (*n*)	34.5 (128)	41.1 (163)
Secondary/high school % (*n*)	33.4 (124)	50.9 (202)
University % (*n*)	28.0 (104)	7.1 (28)
Type of housing		
Ordinary % (*n*)	100 (371)	90.4 (359)
Special % (*n*)	0 (0)	9.6 (38)
Type of dwelling		
Multi-dwelling block % (*n*)	59.3 (220)	83.1 (330)
One-family house % (*n*)	40.7 (151)	16.9 (67)
Geographical area		
Urban % (*n*)	74.1 (275)	83.4 (331)
Semi-urban % (*n*)	22.9 (85)	13.6 (54)
Rural % (*n*)	3.0 (11)	3.0 (12)
Years in present dwelling md (q1-q3)	16 (6–32)	19 (7–32)

### Instruments

The core set of measures was the same for the two original studies from which the data used was generated, which allows us to make direct comparisons on specific variables. Demographic variables included age, sex, marital status, education, type of housing (ordinary or special), type of dwelling (multi- or single-family), type of geographic location (rural, semi-urban, urban), and years in the present home. The demographic variables were used to describe the two cohorts. Details about ENABLE-AGE and the Home and Health in the Third Age Study have been published elsewhere [11, 12].

### Objective aspects of home

The number of environmental barriers and magnitude of accessibility problems were captured with the Housing Enabler (HE) instrument [15]. The HE is administered by a trained rater who objectively assesses the home environment (indoors, at entrances and immediate exterior surroundings) for environmental barriers that could cause accessibility problems for the inhabitant. The participants were also objectively assessed for their functional limitations, including use of mobility devices as an indicator of more severe mobility problems. Based on the combination of functional limitations and environmental barriers present in the home, an accessibility score was calculated. Higher scores are indicative of more accessibility problems, with a score of zero indicating no such problems. People with no functional limitations will have an accessibility score of zero regardless of the number and character of environmental barriers in their homes. The theoretical maximum score is 1,844, but it is not likely to occur; in the sample from the ENABLE-AGE project, the highest score was 670.

### Perceived aspects of home

The Usability in My Home questionnaire (UIMH) was used to capture the degree to which the physical environment is perceived to support performance of daily activities in the home [16, 17]. We used two sub-scales that target aspects of activity (4 items) and aspects of the physical environment (6 items) on usability. The items are rated on a five-point scale ranging from 1 (not at all suitable/usable) to 5 (fully suitable/usable); higher scores mean higher usability. The 10-item scale reached a good level of internal consistency, Cronbach’s *α* = 0.84. We used the total score from all 10 items (range = 10–50) as well as categories based on quartiles.

The 28-item Meaning of home (MOH) questionnaire captures behavioral (6 items), physical (7 items), cognitive/emotional (10 items) and social (5 items) aspects. Each item is rated on a scale ranging from 0 (strongly disagree) to 10 (strongly agree). Higher scores indicate a stronger bonding/attachment to home [18]. The 28-item scale (range = 0–280) reached an acceptable level of internal consistency, Cronbach`s *α* = 0.78. We used the total score from the 28-item scale and categories based on quartiles.

External control beliefs in relation to home were addressed using the Housing-related Control Beliefs Questionnaire (HCQ). External control indicates that an external power such as another person is responsible, or that things happen by plain luck, chance, or fate. In order to capture such aspects, data on the two factors External Control: Powerful Others (8 items) and External Control: Chance (8 items) were combined into a 16-item scale (range = 16–80) [19]. Each item was assessed on a five-point rating scale ranging from 1 (do not at all agree) to 5 (agree very much); higher scores indicate lower perceived external control [20]. This scale reached an acceptable level of internal consistency, Cronbach`s *α* = 0.69. We used the total score based on quartiles.

Housing satisfaction was assessed with the single question “*Are you happy with the conditions of your home*?” adapted from the “Housing Options for Older People” (HOOP) Questionnaire A five-point rating scale ranging from 1 (no, definitely not satisfied) to 5 (yes, definitively satisfied) was presented to the participants (Sixsmith and Sixsmith, unpublished ENABLE AGE working paper, 2002) [21].

### Aspects of health

The Ryff scales of Psychological Wellbeing (PWQ) [22] incorporate several different theoretical perspectives and measure positive psychological functioning. A short form with 18 items divided in two domains, autonomy (9 items) and purpose in life (9 items), was used. Statements were presented to the participants with the instruction to rate each statement on a scale ranging from 1 (strong disagreement) to 5 (strong agreement). Examples of statements are, “*I am not afraid to voice my opinions even when they are in opposition of most people*” (autonomy), and “*Many daily activities often seem trivial and unimportant to me*” (purpose in life). Some items are negatively phrased and need to be reversed when computing sum scores. Scores are computed for each domain; a high score indicates a higher feeling of mastery. A sum score of both domains gives an indication of overall psychological well-being. Cronbach’s alpha in our dataset indicated acceptable internal consistency [23], purpose in life *α* = 0.65 and autonomy *α* = 0.71.

To capture depressive symptoms, the 15 item version of the Geriatric Depression Scale (GDS-15) was used [24]. For each item, the participant was asked to answer yes or no based on how he/she had felt over the past week. Five items indicate a depressive symptom when rated no, while the remaining 10 items indicate a depressive symptom when rated yes. Each “depressive” answer equals 1 point, with possible scores ranging from 0–15. Cronbach’s alpha in our dataset indicated acceptable internal consistency; *α* = 0.77.

A checklist consisting of 30 items was used to dichotomously (yes/no) assess the presence of symptoms in seven different domains: depression (exhaustion, sleep disturbance, general fatigue, depression and cries easily), tension (restlessness, difficulty in relaxing, impaired concentration, nervousness and irritability), gastrointestinal-urinary (abdominal pain, constipation, diarrhea, nausea, anorexia, difficulty passing urine), musculoskeletal (pain in the legs, backache and pain in the joints), metabolism (overweight, loss of weight, sweating and feeling cold), heart-lung (cough, chest-pain and breathlessness), head symptoms (dizziness, headache, impaired hearing and eye problems). Each participant was asked to answer yes when he/she had experienced a symptom during the past three months [25].

Perceived functional independence (PFI) was addressed by the question “All in all, how would you evaluate your own independence, i.e. in performing activities of daily living?” scored from 0 (completely dependent) to 10 (completely independent); only the endpoints were defined.

The total number of functional limitations was calculated from the HE instrument. The presence of limitations (yes/no) was counted with possible scores ranging from 0 to 12. In addition, as an indicator of more severe mobility limitations, use of mobility devices (indoors and outdoors) was assessed (in use/not in use) from the three categories cane/crutch, walking frame and wheelchair [10].

The ADL Staircase was used to assess dependence in activities of daily living (ADL), including five items of personal ADL (PADL; feeding, transferring, toileting, dressing, bathing) and four instrumental ADL items (IADL; cooking, transportation, shopping, cleaning). The instrument was administered using a combination of interview and observation. The assessment was recorded on a three-point scale (independent, partly dependent and dependent), with dependence defined in terms of assistance from another person. After dichotomization based on the principles stated in the original instrument manual [26], the ADL Staircase was used to summarize a participant’s overall ADL ability with the degree of dependence ranked from 0 (independent in all activities) to 9 (dependent in all activities). The ADL Staircase is reliable and valid for the assessment of older people’s functional abilities [27]. In addition to the ADL Staircase, a question on self-perceived difficulty in ADL was used to capture the heterogeneity of ADL capacity within the group of participants who were rated as independent. Directly after a participant was rated as independent in an ADL Staircase item, the participant was asked whether he/she performed the specific task with or without difficulty.

### Data analysis and statistical methods

Differences in proportions (domains of symptoms, use of mobility devices, ADL and depression above cut off ≥5 points) were tested with the Pearson’s Chi-squared test (Table 3).

For all other categorical variables (objective and perceived aspects of home, psychological well-being, depression, number of symptoms, perceived functional independence and functional limitations) Kolmogorov-Smirnovs test was applied to control for normality in the respective age cohort. All variables, with the exception of “total number of barriers” in the younger old cohort, showed a skewed distribution (K-S, *p <* 0.05). Thus, based on type of scale and distribution results are given as medians and inter-quartile ranges; differences between age cohorts were tested non-parametrically using the Mann–Whitney U test. P-values were corrected according to Bonferroni’s method. The corrections were done taking into account all made tests (Tables 2 and 3). All analyses were two-sided and the level of significance was set to *p <* 0.05. Analyses were performed using SPSS software version 21 (IBM Corporation, Armonk, NY, USA).

**Table 2:** Comparison of objective and perceived aspects of home between the younger old and very old cohort

Aspect of home variable	Younger old cohort *N =* 371	*N*	Very old cohort *N =* 397	*N*	*p*-value^g,h^
Objective aspect					
No. of environmental barriers^a^					
Outdoors, md (q1-q3)	9 (7–12)	370	10 (8–13)	396	0.004
Entrances, md (q1-q3)	13 (9–19)	370	12 (8–15)	396	0.019
Indoors, md (q1-q3)	45 (41–50)	370	35 (31–45)	396	<0.001
Total, md (q1-q3)	69 (62–76)	370	58 (49–66)	396	<0.001
Accessibility score^b^					
Outdoors, md (q1-q3)	0 (0–10)	370	30 (6–55)	389	<0.001
Entrances, md (q1-q3)	0 (0–7)	370	21 (3–44)	389	<0.001
Indoors, md (q1-q3)	0 (0–34)	370	46 (13–80)	389	<0.001
Total, md (q1-q3)	0 (0–58)	370	99 (37–184)	389	<0.001
Perceived aspect					
Usability of the home^c^					
Activity md (q1-q3)	20 (19–20)	368	20 (18–20)	382	0.289
Environmental md (q1-q3)	29 (26–30)	371	28 (26–30)	383	0.777
Meaning of home^d^					
Physical md (q1-q3)	61 (55–66)	368	64 (59–70)	384	<0.001
Behavioral md (q1-q3)	54 (48–59)	368	53 (45–60)	385	1.0
Cognitive-emotional md (q1-q3)	85 (78–92)	368	86 (79–91)	384	1.0
Social md (q1-q3)	47 (41–50)	368	45 (40–50)	387	0.027
Housing related control beliefs^e^					
External control md (q1-q3)	36 (31–41)	369	45 (40–51)	373	<0.001
Housing satisfaction^f^ md (q1-q3)	5 (5–5)	371	5 (5–5)	387	1.0

Due to internal dropout in the variables used, in the younger older cohort n varies from 368–371, and in the very old cohort from 373–396

^a^No. of environmental barriers outdoors 28 items, entrance 46 items, indoors 87 items

^b^Accessibility; higher score = less accessibility

^c^Usability of the home: activity; 4 items, score range 4–20, higher = better usability; environment: 6 items, score range 6–30, higher = better usability

^d^Meaning of home; for all subscales a higher score indicates a stronger bonding, Physical; 7 items score range 0–70, Behavioral 6 items, score range 0–60, Cognitive-emotional: 10 items score range 0–100, Social 5 items, score range 0–50

^e^Housing related control external: 16 items, score range 16–80, higher score = more external control

^f^Housing satisfaction: 1 item, score range 1–5, higher = more satisfied

^g^Diferences in medians were tested using the Mann–Whitney

^h^ *P*-values corrected according to the Bonferroni method

**Table 3:** Aspects of health: Comparison of psychological well-being, depression, symptoms, perceived functional independence, functional limitations, use of mobility devices and ADL between the younger old and the very old cohort

Aspects of health variable	Younger old cohort *N =* 371	*N*	Very old cohort *N =* 397	*N*	*p*-value^e,f,g^
Psychological well-being^a^					
Autonomy md (q1-q3)	34 (31–38)	370	35 (31–38)	378	1.0
Purpose in life md (q1-q3)	32 (29–36)	368	27 (24–30)	381	<0.001
Depression^b^					
Mood md (q1-q3)	0 (0–1)	369	1 (0–2)	394	<0.001
Motivation md (q1-q3)	0 (0–1)	370	2 (1–3)	395	<0.001
Total score md (q1-q3)	1 (0–2)	369	3 (1–4)	394	<0.001
≥ 5 points % (*n*)	9.2 (34)		20.4 (81)		<0.001
Symptoms, total no.md (q1-q3)	5 (3–10)	361	7 (4–10)	384	0.126
Depression % (*n*)	61.2 (227)		67.0 (266)		1.0
Tension % (*n*)	44.5 (165)		46.2 (182)		1.0
Gastro-urinary % (*n*)	40.8 (150)		50.6 (200)		0.252
Musculoskeletal % (*n*)	67.4(250)		75.3 (298)		0.669
Metabolism % (*n*)	42.8 (169)		53.0 (195)		0.210
Heart-lung % (*n*)	45.8 (170)		47.9 (190)		1.0
Head % (*n*)	62.5 (232)		81.3 (321)		<0.001
Perceived func. indep. md (q1-q3)	10 (9–10)	370	9 (8–10)	385	<0.001
Functional limitations md (q1-q3)	1 (0–2)	370	2 (1–4)	389	<0.001
Mobility device use					
Indoors					
Cane/crutch % (*n*)	1.4 (5)		12.8 (51)		<0.001
Walking frame % (*n*)	1.6 (6)		11.8 (47)		<0.001
Wheelchair % (*n*)	0.3 (1)		0.8 (3)		1.0
Outdoors					
Cane/crutch % (*n*)	3.5 (13)		29.5 (117)		<0.001
Walking frame % (*n*)	3.0 (11)		26.4 (105)		<0.001
Wheelchair % (*n*)	0.3 (1)		3.8 (15)		0.028
Activities of daily living					
Independent without difficulty % (*n*)	78.9 (292)		16.7 (63)		<0.001
Independent with difficulty % (*n*)	9.5 (35)		18.3 (69)		0.036
Dependent IADL^c^ % (*n*)	11.1 (41)		54.4 (205)		<0.001
Dependent IADL and PADL^d^ % (*n*)	0.5 (2)		10.6 (40)		<0.001

Due to internal dropout in the variables used, in the younger older cohort n varies from 361–371, and in the very old cohort from 378–395

^a^Psychological well-being: Autonomy 9 items, score range 9–45, higher score = higher feeling of mastery; Purpose in life 9 items, score range 9–45, higher = higher feeling of mastery

^b^Depression: Mood 10 items, score range 0–10, higher = more depressive; Motivation 5 items, score range 0–5, higher = less motivated

^c^IADL = Instrumental Activities of Daily Living

^d^PADL = Personal Activities of Daily Living

^e^Differences in proportions were tested with the Pearson’s Chi-squared test

^f^Differences in medians were tested with the Mann–Whitney U test

^g^P-values corrected according to Bonferroni’s method

### Ethics approval and consent to participate

The ENABLE-AGE Study, the Home and Health in the Third Age Study and the SNAC-GÅS Project were conducted in accordance with the Helsinki Declaration [28]. Informed consent was obtained from all participants and confidentiality was ensured. Participants could withdraw from the study whenever they wished. Following the legislation and procedures for formal ethical approval valid at the time of the respective project start, the ENABLE-AGE Study and the SNAC-GÅS Project were approved by the Ethics Committee of Lund University, Sweden (reg. nos. LU 842–02, LU 744–00), and the Home and Health in the Third Age Study was approved by the Ethical Board in Lund (reg. no. 2010/431).

## Results

### Objective aspects of home

Comparing the younger old and the very old cohorts, the median of the total number of objectively assessed environmental barriers was 69 (q1 = 62, q3 = 76) and 58 (q1 = 49, q3 = 66) (U [370, 396] =34 128, *p <* 0.001) respectively, showing that those in the very old cohort lived in housing with fewer environmental barriers. While the total number of environmental barriers was significantly different between the two cohorts, the numbers in exterior surroundings and at entrances did not differ. Still, the very old cohort lived in dwellings with more accessibility problems (median total score = 99; q1 = 37, q3 = 184), than the younger old cohort (median total score = 0; q1 = 0, q3 = 10), (U [389, 370] =33 249, (*p <* 0.001). The largest difference in accessibility between the two cohorts was found indoors (Table 2).

### Perceived aspects of home

Physical and social aspects of meaning of home showed significant differences between the two cohorts. The median for the physical aspects was somewhat lower; 61 (q1 = 55, q3 = 66), in the younger old cohort compared to 64 (q1 = 59, q3 = 70), (U [368, 384] =55 156, *p <* 0.001) in the very old cohort. That is, in comparison with the very old cohort, the younger old experienced the conditions of their homes as less favorable. For social aspects though, the situation was the opposite; the median was higher in the younger old cohort, 47 (q1 = 41, q3 = 50) compared to 45 (q1 = 40, q3 = 50), (U [368, 387] =61 198, *p =* 0.027 in the very old cohort. Thus, the younger old cohort found their homes more suitable for social contacts with friends or people in the neighborhood than the very old cohort did. The activity and environmental aspects of usability of the home showed no differences in medians even though significance was reached due to the wider distribution of scores in the younger old cohort (Table 2).

### Aspects of health

Participants in the very old cohort had more functional limitations, median score 2 (q1 = 1, q3 = 4), compared the younger old cohort, median score 1 (q1 = 0, q3 = 2), (U [389, 370] =30 361, *p <* 0.001), reported lower perceived functional independence, median score 10 (q1 = 9, q3 = 10) compared to, median score 9 (q1 = 8, q3 = 10), (U [385, 370] =50 383, *p <* 0.001), and were more likely to use mobility devices (indoors as well as outdoors), compared to the younger old cohort (Table 3). Dependence in at least one ADL was reported by 65.0 % of the participants in the very old cohort and by 11.6 % of the younger old cohort (χ^2^ [245, 43] = 224, *p <* 0.001). The very old cohort also reported a larger number of symptoms, even though not significant, median score 7 (q1 = 4, q3 = 10) than the younger old cohort, median score 5 (q1 = 3, q3 = 10), (U [384, 361] = 60 526, *p =* 0.126). Among the domains of symptoms, those from head including vision-, hearing impairment, dental symptoms and headache, showed the largest differences between the two cohorts (Table 3).

## Discussion

With the present study, differences in the home and health situation between two sub-groups of the ageing population in southern Sweden were identified. While the two cohorts were recruited based on age criteria and thus represent a very old and a younger old cohort, respectively, it should be kept in mind that they differed also in other respects that deserve further research attention. Still, since the differences in health were in line with previous research based on age differences [25, 29], the results regarding objective and perceived aspects of home indicate intriguing differences that represent new insights that deserve further research attention.

Although the very old cohort lived in dwellings with fewer environmental barriers, in particular indoors, they still had more accessibility problems due to having more functional limitations. Therefore, even if people seem to seek “better housing” as they age, they do not sufficiently compensate for the increasing complexity of functional limitations coming with advanced age [29]. Accordingly, foresighted planning as well as the availability of ordinary housing without the types of environmental barriers that typically generate accessibility problems for very old people may be crucial if one can stay in ordinary housing or have to move to special housing [3, 30, 31].

Further, previous results based on data on the very old cohort have shown that environmental barriers are more prevalent in multi-dwelling blocks compared to one-family housing, and that about 80% of the environmental barriers are related to the indoor environment or entrances [12]. Taking into account that over 83% of the very old cohort lived in multi-dwelling blocks, the ageing population places great demands on public and private providers of such housing to reduce the most common and problematic environmental barriers and thereby reduce the occurrence of the accessibility problems. However, increasing the knowledge further on such details of the housing environment is a more complex matter than might be expected as differences in terms of specific environmental barriers are not only dependent on type of housing but very much so also on building period [3]. Besides the fact that such detailed analyses would increase the complexity of the present study to an extent that would motivate a study in its own right, unfortunately we do not have access to building year for the younger cohort. Still, with such evidence at hand, research could serve housing providers with useful information for refurbishment as well as new housing projects.

Overall, the younger old cohort is also in a better situation related to perceived aspects of housing (see Table 2). Although the differences are small for several variables, they are still significant for most aspects. The most pronounced difference between the two cohorts studied is related to external housing related control believes. As less influence over the housing situation has been shown to be associated with greater accessibility problems as well as dependence in ADL [32], this is an important finding with implications for the home and health situation of very old, single-living community-dwelling people. Consistent with the ETA [8, 9], the more favorable situation in usability for the younger old cohort reflects the fact that in an earlier phase of the ageing process people might have the capacity to maintain adaptive behavior even in situations exerting higher environmental press. Still, the participants in the very old cohort were as satisfied with their housing situation as those of the younger old. Keeping in mind the weakness of our study in terms of limited comparability between the two cohorts, we argue that the results demonstrate that in-depth investigations of different aspects of housing are necessary to increase the knowledge about the complex dynamics of home and health among different sub-groups of the ageing population [10]. This complexity is further demonstrated by the mixed picture of differences regarding meaning of home between the two cohorts. The participants in the younger old cohort were in a more positive situation in terms of behavioral and social meaning of home, while those of the very old cohort rated physical and cognitive/emotional domains more favorably. As to the latter, this might reflect a stronger bonding to home based on familiarity and habits which in turn might counteract the willingness and ability to take action to change the housing situation. However, based on one single study with limited comparability between the two cohorts studied this is sheer speculation, but intriguing and worth further research. All in all, as elucidated in a recent study residential reasoning in very old age is a complex and ambivalent matter [33], and professionals as well as family and older people themselves should be made aware of the need for increased attention to not only objective but also to perceived aspects of housing in counselling and intervention planning targeting older people.

During normal ageing, physical, mental and cognitive abilities as well as the ability to resist diseases decrease, which in turn increases the risk of symptoms and morbidity [25, 34]. Hence, it was not unexpected that a larger proportion or participants in the very old cohort had more symptoms in each domain even though only the domain of head symptoms reached significance, (see Table 3). Still, only 4.5 % in the younger old cohort reported no current symptoms; the corresponding proportion in the very old cohort was 2.3 %. The largest difference was found in the domain of head symptoms, where a fourth of the younger old cohort and about half of the very old cohort responded that they had eye problems. This is worth noting because vision impairment by itself can lead to accessibility problems and ADL dependence [35].

Depression is common among older adults and the incidence and prevalence increase with age [36]. According to a review by Djernes et al. published in 2006 [37], the prevalence of depression among older people living in ordinary housing or institutional settings varied from 7.2 % to 49.0 %. This large difference shows how difficult it can be to diagnose a real depression due to different methods and definitions. In our study using the GDS-15 scale, we found a higher prevalence of suspect depression in the very old cohort, which is consistent with previous findings [38]. For symptoms of depression [25] a higher proportion in the very old cohort reported higher numbers even though not significant compared with the younger old cohort, suggesting the difficulty in recognizing symptoms of a depression among very old people. However, once again the limited comparability between the two cohorts should be noted, but since the differences in aspects of health were in the expected direction we regard the new insights indicating differences in aspects of home should as valid and useful as a starting-point for further studies in this field of inquiry.

Since we had access to uniquely detailed data on objective and perceived aspects of housing collected with a younger old and a very old cohort, we used existing databases for this first, descriptive study to shed light on the situation of two different sub-groups of the ageing population. Naturally, a subdivision by chronological age includes a large group of people with marked individual variation as regards ageing as such. Another way to categorize people along the ageing process is to use Baltes & Smith’s definition of the third and the fourth age [39]. The third age is a part of life after retirement which is characterized by economic security and without major limitations in activities caused by illness or disability. In contrast, the fourth age is the period of life when diseases and disabilities put limits on what an individual can or cannot manage to do, followed by increasing dependence on others to cope with daily activities [40]. Accordingly, it might be more relevant to compare the two cohorts using the definitions of the third and fourth age. That is, out of 12 functional limitations about 50 % of the participants in the younger old cohort answered that they had one or more functional limitations, while on the other hand 10 % in the very old cohort stated that they had none (data not shown). Further, there were two individuals in the younger old cohort that were dependent in IADL as well as PADL, that is, per definition they were in the fourth age. The other way round, in the very old cohort 63 (17 %) of the participants were independent in ADL without difficulty and should accordingly be categorized as belonging to the third age (Table 3). These observations do not only highlight that chronological age might be questioned as the sole inclusion criterion in comparative studies of sub-groups of the ageing population. They also pinpoint the necessity of individualization regarding health promotion, prevention and interventions targeting the home and health situation of people in different phases of the ageing process.

Besides the weakness in terms of limited comparability between the two cohorts studied, there is a risk that the participation rate (i.e., 56 % in the younger old cohort and 45 % in the very old cohort) could jeopardize the external validity. Consequently, it is likely that the participants, especially those in the very old cohort, were healthier than average in the population. Further, the results could be somewhat biased also due to cohort effects, not just because of the age difference but because there was 7 years between data collection points. As to the data on objective aspects of housing, a noteworthy strength is that the dwellings studied are representative of the national housing stock in Sweden [3].

## Conclusion

Keeping in mind that this study was based on data collected with two sub-groups of the ageing population with cohort differences beyond that of age, despite fewer objectively assessed environmental barriers in their dwellings the very old community-living cohort studied lived in housing with more accessibility problems compared to those of the younger old cohort. This difference is caused by the higher prevalence of functional limitations in the very old cohort. Those in the very old cohort perceived themselves in a less favourable situation, but still as satisfied with housing as those in the younger old cohort. This kind of knowledge is indicative for prevention and intervention in health care and social services as for housing provision and societal planning, but further studies based on truly comparable cohorts are warranted.

### Availability of data and materials

The dataset supporting the conclusions of this article is available on request to the corresponding author, Henrik Ekström (henrik.ekstrom@med.lu.se).

## Abbreviations

ADL: activities of daily living
MOH: meaning of home
HCQ: housing related control beliefs
UIMH: usability in my home
P-E-fit: person-environment fit
HOOP: the Housing Option for Older People
SNAC: Swedish national study on ageing and care
